# Case report: Drug reaction with eosinophilia and systemic symptoms (DRESS) induced by ceftazidime in a connective tissue disease (CTD) patient

**DOI:** 10.3389/fphar.2024.1403390

**Published:** 2024-08-12

**Authors:** Rui Dai, Ziran Niu, Yang Yang, Xin Liu, Bo Zhang

**Affiliations:** ^1^ Department of Pharmacy, Peking Union Medical College Hospital, Chinese Academy of Medical Sciences and Peking Union Medical College, Beijing, China; ^2^ Clinical Pharmacology Research Center, Peking Union Medical College Hospital, Chinese Academy of Medical Sciences and Peking Union Medical College, Beijing, China; ^3^ State Key Laboratory of Complex Severe and Rare Diseases, Peking Union Medical College Hospital, Beijing, China

**Keywords:** drug reaction with eosinophilia and systemic symptoms (DRESS), ceftazidime, connective tissue disease, HLA-B∗15:02, case report

## Abstract

Drug reaction with eosinophilia and systemic symptoms (DRESS) is a rare and severe cutaneous adverse drug reactions (SCARs) with high mortality. Antibiotics are the most frequent causative agents related to DRESS. However, it is rarely reported in cephalosporins, especially for ceftazidime. Here, we reported a case of ceftazidime-induced DRESS with HLA genotypic polymorphism as a risk factor. A 58-year-old woman with connective tissue disease was intravenously infused with ceftazidime for the treatment of pneumonia and intestinal infection, followed by the presence of fever, rash, and hematologic and hepatic laboratory abnormalities. DRESS was diagnosed and the positive polymorphism in HLA-B*15:02 was found. Our case illustrated the necessity to clarify the patho-mechanism and the use of pretreatment HLA analysis to prevent ceftazidime-related DRESS may be a valuable option soon.

## Introduction

Drug reaction with eosinophilia and systemic symptoms (DRESS), also known as Drug-induced hypersensitivity syndrome (DiHS), is a life-threatening multi-organ system reaction with a high mortality reaching 10%–40% (Sousa et al., 2016). It can be induced by drugs and is associated with sequential reactivation of herpesviruses (Shiohara and Kano, 2017). Given the rarity and the atypical presentation as an adverse drug reaction (ADR), the incidence of DRESS is obscure. It is previously estimated that the incidence is more than 1 case in 1,000–10,000 prescriptions of each causal drug (Picard et al., 2013; Descamps and Ranger-Rogez, 2014; Shiohara and Kano, 2017). The patho-mechanisms of DRESS are confused with drug exposure, viruses, and immune system factors (Cho et al., 2017), and culprit drug exposure is the primary factor. Withdrawal of the potentially causative drugs immediately and a multidisciplinary regimen is the main treatment strategy for DRESS (Cho et al., 2017).

Aromatic anticonvulsants, allopurinol, and antibiotics are the most frequent causative agents to DRESS (Shiohara and Kano, 2017; Sharifzadeh et al., 2021; Sim et al., 2019). As reported in a review, 254 cases of antibiotic-induced DRESS, antituberculosis, vancomycin, and sulfonamides are the major culprits of antibiotic (Sharifzadeh et al., 2021). Only 10 cases in the review were reported related to cephalosporins including cefotaxime, ceftriaxone, cefadroxil, and ceftazidime (Picard et al., 2013). Through literature research, we found that ceftazidime-induced DRESS was rarely reported.

In previous reports, factors of DRESS eruption included culprit drug exposure, activity of drugs metabolizing enzyme, HLA genetic factors (Tetart et al., 2014), renal or hepatic insufficiency (Shiohara and Kano, 2017), and overwhelm drug dose (Mylonakis et al., 1999). Age or sex had no predilection (Shiohara and Kano, 2017). The polymorphism in genes encoding HLA molecules is a common acceptable prediction factor to prompt drug-induced hypersensitivity (Cho et al., 2017). Such as the HLA-B*13:01 and HLA–A*31:01 allele were associated with carbamazepine-induced DRESS, and the HLA-B*58:01 allele was related to allopurinol-induced DRESS (Ozeki et al., 2011; Hung et al., 2006; Hung et al., 2005). Of note, for lots of culprit drugs, a genetic predisposition for patients with DRESS is still unidentified. Herein, we reported the second rare case of DRESS induced by ceftazidime in a connective tissue disease (CTD) patient, to improve the awareness of DRESS risk in the immunosuppression population with the treatment of third-generation cephalosporins. Furthermore, HLA gene sequencing was performed in this case and the potential significance was analyzed.

## Case summary

A 58-year-old woman was diagnosed with CTD and complicated by interstitial pneumonia and gastrointestinal disorder. The diagnosis of CTD was based on the presence of a marked febrile (body temperature of 39°C), photosensitivity, and Raynaud’s phenomenon. Abnormal biochemical indicators with white blood cell count (WBC) 3.90 × 10^9^/L, neutrophils 83.6%, mild liver enzymes elevated with alanine aminotransferase (ALT) 53.8 U/L, aspartate transaminase (AST) 41.7 U/L, elevated inflammatory markers with positive antinuclear antibody (ANA) and Epstein-Barr virus (EBV). But no signs of organ involvement in the early stage. A combination of leflunomide, hydroxychloroquine, and prednisone tablet was administrated. The daily dose of prednisone was decreased gradually from 60 to 12.5 mg.

Five months later, with CTD progression and side effects from long-term use of glucocorticoids, the patient had pneumonia disorder, multiple ulcers of the colon with digestive tract infection, and oral mucosal erosion. For colonic ulcers, increased doses of oral prednisone to 30 mg once a day with mesalazine, mycophenolate mofetil, and thalidomide were administered. Several drugs including ceftazidime, tinidazole, fluconazole, and piperacillin tazobactam were prescribed for the treatment of pneumonia and digestive infection. During this treatment stage, the patient developed a fever (38.5°C) and erupted rash characterized by exfoliative, congestive, and diffused from the navel to the whole body. Part of the skin is pigment deposition with desquamation. The physician suspected drug-induced atopic dermatitis (AD) and prescribed an antiallergic regime with a combination of loratadine and topical cream (compound ingredients of miconazole, triamcinolone acetonide, and neomycin) at first. However, the rash did not improve.

**FIGURE 1:**
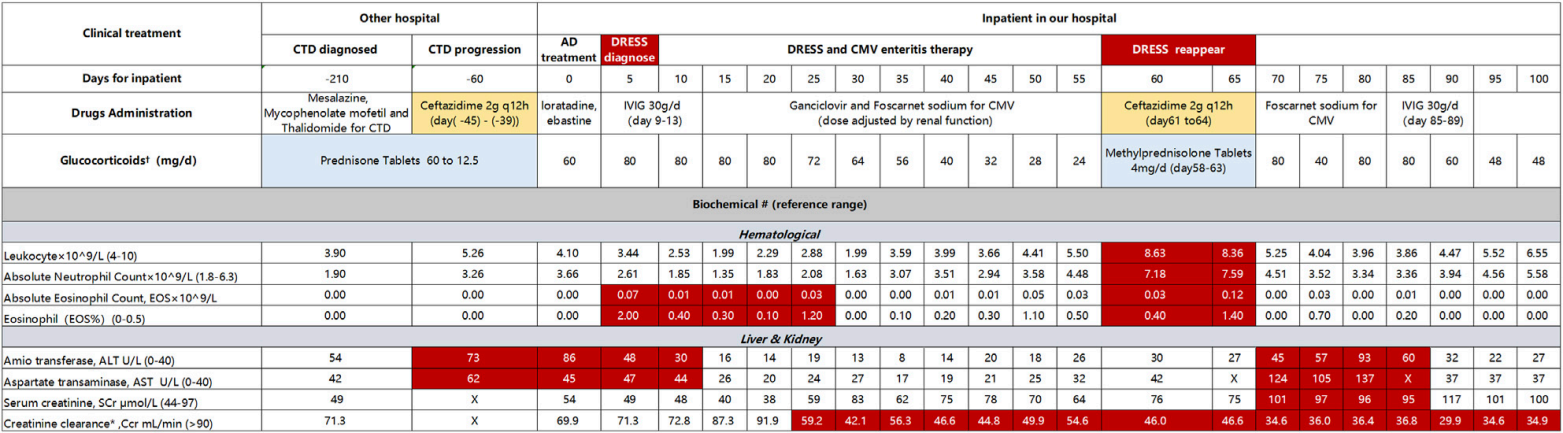
Clinical treatment and laboratory testing indicators CTD, connective tissue disease; AD, atopic dermatitis; IVIG, intravenous immunoglobulin. ^†^Blank block means Methylprednisolone for injection. ^#^Red block indicated abnormal value, X means no detection. *Creatinine clearance (Ccr) calculated by formula of Cockcroft-Gault (Cockcroft and Gault, 1976), CCr = [(140-year) × weight)/(72 × Serum creatinine (umol/L)] × 0.85 (Patient: Female, 58 years, 41 kg).

About 30 days later, the patient was hospitalized in our hospital (45 days after ceftazidime administration). The maculopapular rash was shown obviously. Rash involving > 50% of body surface area, diffuse erythema in the whole body, and psoriasiform desquamation in arms and legs with positive Nikolsky’s sign. Facial and four limbs were edema. No palpable swelling of superficial lymph nodes. Laboratory testing demonstrated hematologic, hepatic, and pathogen abnormalities (Figure 1), which included leukocytosis (leucocytes from 3.9 to 5.26 × 10^9^/L), eosinophilia (absolute eosinophil (EOS) count from 0 to 0.07 × 10^9^/L), lymphadenopathy, ALT and AST mild elevated to 72.9 and 61.8 U/L, respectively. Cytomegalovirus (CMV) was positive (copies of CMV-DNA are 59,000/mL, reference range < 500 copies/mL), but EBV, human herpesvirus 6 (HHV6/7), and hepatitis virus were all negative. According to the diagnostic criteria for DRESS defined by the International RegiSCAR-group (Kardaun et al., 2013; Sasidharanpillai et al., 2022; Mansour et al., 2023), DRESS was considered to be definite with a score of 7 points (Table 1). For progressive DRESS and CMV enteritis, the daily dose of methylprednisolone decreased from 80 to 24 mg intravenous (dose adjusted for creatinine clearance) and transfusion for CMV enteritis-induced gastrointestinal hemorrhage. About 2 weeks later, the rash and gastrointestinal of the patient were stable, and sequential therapy was engaged with oral methylprednisolone.

**TABLE 1:** RegiSCAR scoring of the patient.

Score	−1	0	1	2	Case score
Fever ≥ 38.5°C		Yes			0
Enlarged lymph nodes		No			0
Eosinophilia		No			1
Eosinophils			100–1,200/μL		
Eosinophils, if leukocytes < 4,000		0.1%–2.0%			
Atypical lymphocytes			Yes		1
Skin involvement					2
Skin rash extent (% BSA)			>50%		
Skin rash suggesting DRESS			Yes		
Biopsy suggesting DRESS		Unknown			
Organ involvement^a^					2
Liver			Yes		
Kidney			Yes		
Lung			Yes		
Muscle/heart		No			
Pancreas		No			
Other organ(s)		No			
Resolution ≥ 15 days		Yes			0
Evaluation other potential causes					1
ANA		Negative			
Blood culture		Negative			
Serology for HVA/HVB/HVC Chlamydia-/*Mycoplasma* pneumoniae		Negative			
Other serology/PCR		Negative			
If none positive and ≥ 3 of above negative			Yes		
TOTAL SCORE					7

^a^ After exclusion of other explanations: 1 = 1 organ, 2 = ≥ 2 organs.

The patient had hormone dependence on DRESS. When reducing the dose of glucocorticoids, rash and CMV enteritis erupt again, and the indicators shown deteriorate (Figure 1). Specifically, leucocytes increased from 3.39 to 8.63 × 10^9^/L, absolute neutrophil count increased from 3.07 to 7.59, absolute EOS 0.12 × 10^9^/L, ALT and AST increased from 6 and 20 U/L to 93 and 137 U/L, respectively. Copies of CMV-DNA are 9,370/mL. At the same time, pulmonary infection aggravated, and ceftazidime (2g q12h) was prescribed again for 4 days. However, the pneumonia did not improve. The body temperature sharply increased to 38°C, and the rash deteriorated. Ceftazidime was stopped immediately and changed to sulfamethoxazole-trimethoprim (TMP-Co) with ertapenem because of the identification of mixed infection by Pneumocystis carinii pneumonia (PCP) and *Escherichia coli* producing extended-spectrum beta-lactamases (ESBL- *E. coli*). The total treatment had up to 100 days, the patient manifested respiratory failure and DRESS was not in complete remission. Because of the poor prognosis and expensive cost, the patient was discharged.

## Detecting of HLA genetic polymorphism

The study protocol was approved by the medical ethics committee of Peking Union Medical College Hospital. After the patients provided the written informed consent, we collected peripheral blood samples from 2 mL of the patient to take HLA gene sequencing.

Genomic DNA extraction from peripheral blood samples was performed using the EasyPure Blood Genomic DNA Kit (TransGen Biotech) and quantified using agarose gel electrophoresis. In the pilot study, the HLA sequences (chr6:28477797-33448354) were efficiently enriched in 1.0-μg genomic DNA as determined by using SeqCap EZ Choice Enrichment Kits (NimbleGen) according to the manufacturer’s protocol. Fragments between 180 and 220 bp in length were extracted and sequenced using the Illumina HiSeq X Ten system. Two common HLA alleles in Han Chinese were enrolled in our detection, including HLA-B*58:01 and HLA-B*15:02. We got polymorphism in HLA-B*15:02 (HLA-B*15:02TA (C > G) CC and HLA-B*15:02TB (C > T) CT). HLA-B*58:01 is negative (HLA-B*58:01TA(T > G) TT, HLA-B*58:01TC (A > T) AA.

## Discussion

In this report, we elaborated on the second rare case of ceftazidime-induced DRESS. With the collection of HLA genotype samples, we promoted HLA-B*15:02 that has promising a risk factor for ceftazidime treatment in immunodeficiency patients.

### Diagnose of DRESS

DRESS syndrome is characterized by a constellation of symptoms including widespread rash, fever, hematologic abnormalities (such as eosinophilia or lymphadenopathy), and systemic involvement (affecting the liver, kidneys, lungs, heart, pancreas, and other organ damage) (Shiohara and Kano, 2017; Cacoub et al., 2011). The onset of symptoms in DRESS syndrome typically occurs between 2 and 8 weeks after drug administration, and they can persist even after discontinuing the suspected drug, lasting for more than 2 weeks with possible relapses months later (National Institute of Diabetes and Digestive and Kidney Diseases, 2012). Our patient exhibited these typical features of DRESS syndrome and its occurrence in about 6 weeks. Importantly, eosinophilia in DRESS cases can be delayed, occurring up to 1–2 weeks after the initial symptoms, and sometimes even after normalization of liver enzymes (Shiohara and Kano, 2017). This delay significantly adds to the diagnostic challenges associated with DRESS syndrome. In the current case, eosinophilia was observed approximately 4 weeks later, likely due to infrequent testing at other healthcare facilities. Positive markers for EBV, CMV, and HHV are crucial indicators of DRESS, with viral loads reflecting disease severity (Shiohara and Kano, 2017). However, only CMV tested positive in this specific instance, while EBV and HHV remained negative. The reactivation of herpesviruses, triggered by an allergic immune response to medication and subsequent activation of T-cell populations (notably cytotoxic CD8^+^ lymphocytes), can cause direct tissue damage (DeClerck and DeClerck, 2018). The frequent deterioration or recurrent flare-ups of clinical symptoms after withdrawing the causative drugs are likely due to sequential reactivations of herpesviruses. This aligns with our case, where two peaks of CMV-DNA load were observed during the DRESS episode (Shiohara and Kano, 2017).

### Culprit drug adjustment for DRESS

DRESS is a latency reaction, often requiring the administration of multiple medications before a diagnosis can be made, making it difficult to pinpoint the culprit drug. Based on the Naranjo ADR Probability Scale to assess causality between DRESS and drug (National Institute of Diabetes and Digestive and Kidney Diseases, 2012) (Table 2), ceftazidime emerged as the prime suspect in causing DRESS. Before the rash appeared, the patient had been taking seven potential drugs simultaneously for 7 days, which included mesalazine, mycophenolate mofetil, thalidomide, ceftazidime, tinidazole, fluconazole, and piperacillin-tazobactam. During this time, the patient exhibited immediate allergic reactions resembling drug-induced AD, but anti-allergic treatments proved ineffective. Approximately 45 days later, the rash worsened, and DRESS was diagnosed. All seven drugs were promptly withdrawn. Fortunately, the patient’s symptoms improved after receiving glucocorticoids and intravenous immunoglobulin (IVIG) treatment. Although all seven drugs were suspects due to their temporal association with the rash, there are no reported cases linking tinidazole, fluconazole, mesalazine, mycophenolate mofetil, or thalidomide to DRESS. Despite scoring 6 points on the Naranjo scale, indicating a “Probable” cause, we excluded these drugs based on clinical grounds. However, when ceftazidime was re-administered empirically due to a pulmonary infection, DRESS recurred. Prior studies have established that antibacterial antibiotics, especially cephalosporins, are high-risk drugs for DRESS. Sharifzadeh et al. reviewed 254 cases with a definite or probable diagnosis of antibiotic-induced DRESS (Sharifzadeh et al., 2021), of which 10 were attributed to cephalosporins, with only one caused by ceftazidime (Picard et al., 2013; Sharifzadeh et al., 2021). Furthermore, other diseases and alternative drugs were ruled out as potential causes of DRESS. According to the Naranjo scale scored nine points, which provided a “definite” result for ceftazidime (Naranjo et al., 1981).

**TABLE 2:** Association assessment between DRESS and culprit drugs in Naranjo scale.

Question	Yes	No	Do not know	Score						
				Ceftazidime	Piperacillin tazobactam	Tinidazole	Fluconazole	Mesalazine	Mycophenolate mofetil	Thalidomide
1. Are there previous conclusive reports on this reaction?	1	0	0	1	1	0	0	0	0	0
2. Did the adverse event appear after the suspected drug was administered?	2	−1	0	2	2	2	2	2	2	2
3. Did the adverse event improve when the drug was discontinued or a specific antagonist was administered?	1	0	0	1	1	1	1	1	1	1
4. Did the adverse event reappear when the drug was readministered?	2	−1	0	**2**	0	0	0	0	0	0
5. Are there alternative causes that could on their own have caused the reaction?	−1	2	0	2	2	2	2	2	2	2
6. Did the reaction reappear when a placebo was given?	−1	1	0	0	0	0	0	0	0	0
7. Was the drug detected in blood or other fluids in concentrations known to be toxic?	1	0	0	0	0	0	0	0	0	0
8. Was the reaction more severe when the dose was increased or less severe when the dose was decreased?	1	0	0	0	0	0	0	0	0	0
9. Did the patient have a similar reaction to the same or similar drugs in any previous exposure?	1	0	0	0	0	0	0	0	0	0
10. Was the adverse event confirmed by any objective evidence?	1	0	0	1	1	1	1	1	1	1
Total Score				9	7	6	6	6	6	6

**FIGURE 2:**
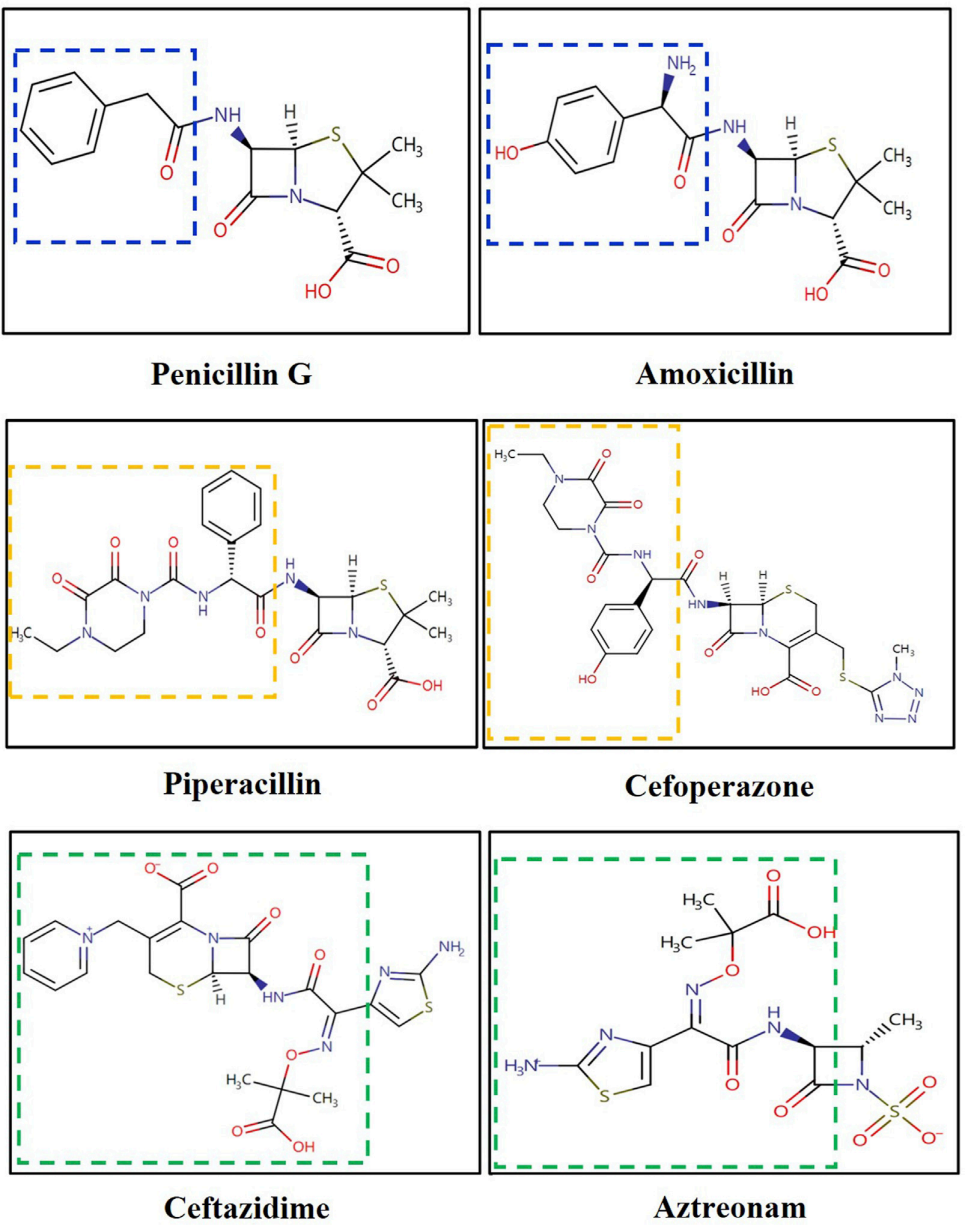
Structure of cephalosporins and penicillins. *Both drugs of same color line have the same or similar side chain.

It is noteworthy that, according to the Naranjo scale, ADR relationships scoring between 5 and 8 points are considered “Probable”. Piperacillin-tazobactam had seven points with high frequency reported (accounting for 50% of cases in Penicillin-induced DRESS (n = 22) (Sharifzadeh et al., 2021). However, a preliminary analysis of cross-reactivity mechanisms led us to exclude it. In IgE-mediated immediate hypersensitivity, cross-reactivity between cephalosporins and penicillins is well-documented, primarily attributed to identical R1 side chains (located at the C7 position of the β-lactam ring) (Mansour et al., 2023). Conversely, in non-immediate hypersensitivity reactions like DRESS, this cross-reactivity is less understood (Mansour et al., 2023). Berot et al. have shed light on cross-reactivity among β-lactam antibiotics in non-immediate severe cutaneous adverse drug reactions (SCARs) (Bérot et al., 2020). Out of 18 amoxicillin-suspected cases, 3 (16.5%) showed cross-reactivity with non-amino-cephalosporins (cefotaxime and ceftriaxone) through patch testing. Romano et al. reported a similar finding, with 3 out of 105 cephalosporin-induced SCARs exhibiting cross-reactivity to amoxicillin (Romano et al., 2012). Buonomo et al. reported among 97 penicillin-induced SCARs, 17.5% had cross-reactivity to cephalosporins (10 cephalexin, 9 cefaclor, and 5 cefuroxime) (Buonomo et al., 2014). Pinho et al. found that only 1 out of 109 amoxicillin-induced SCARs cross-reactivity with cephalosporins (cefotaxime and ceftriaxone) (Pinho et al., 2017). Given these findings, penicillin exhibits higher cross-reactivity with first- and second-generation cephalosporins due to their similar side chains. However, third-generation cephalosporins do not share this trait. Among cephalosporins, only cefoperazone has a similar side chain structure to piperacillin. Ceftazidime, on the other hand, despite having the same side chains as aztreonam, rarely exhibits cross-reactivity with penicillin or even piperacillin (Bérot et al., 2020) (Figure 2). Therefore, we have preliminarily excluded piperacillin-tazobactam as a potential culprit.

### Mechanisms between DRESS and ceftazidime

DRESS is a delayed-type hypersensitivity (DTH), which is not induced by IgE (Shiohara and Kano, 2017). The clinical course of this syndrome is typically protracted, complex, and intertwined with anti-viral immune responses and drug hypersensitivity (Cho et al., 2017). Current evidence shows that DRESS syndrome tends to occur in genetically predisposed persons when they are ingesting one of the inciting drugs (Cho et al., 2017). In this case, the patient had underlying hypersensitivity stemming from CTD immunosuppression and was further aggravated by the offending drugs. The pathomechanisms of DRESS syndrome remain complex and largely unknown. Three non-mutually exclusive models have been proposed to elucidate the interactions between drugs or metabolites and immunological synapses: the hapten/pro-hapten model, the pharmacologic interaction (p-i) model, and the altered peptide repertoire model (Cho et al., 2017). In summary, these models postulate that drugs or their metabolites can bind covalently or non-covalently to major histocompatibility complex (MHC) proteins or T-cell receptors, thus triggering T-cell responses.

### Risk factors of HLA alleles

HLA molecules, which are cell surface glycoproteins, play a pivotal role in presenting endogenous and exogenous antigens to T lymphocytes for recognition and subsequent immune response. These molecules are broadly categorized into two classes: HLA class I and HLA class II (Medhasi and Chantratita, 2022). Genetic variations at the loci encoding HLA genes are associated with drug-induced hypersensitivity reactions through interactions with drugs and HLA molecules (Kloypan et al., 2021). Among various ethnic groups, the frequencies of HLA alleles differ significantly. In Han Chinese patients with DRESS, specific polymorphic alleles have been identified. For instance, HLA-A*32:01 is associated with vancomycin (Wang et al., 2022), HLA-A*31:01 and HLA-A*15:02 with carbamazepine (Kloypan et al., 2021), HLA-B*13:01 with co-trimoxazole (Yang et al., 2014), and HLA-B*58:01 with allopurinol (Mariette et al., 2022). In addition, certain alleles like HLA-DRB1*15:01 (Saper et al., 2022) and HLA-DRB1*15:02 (Saper et al., 2022) had high frequency in Asian populations but only proved in European and African populations, which had prompt value to some extent. Notably, despite the extensive research on HLA alleles and drug-induced adverse reactions, there has been a paucity of studies exploring the link between HLA and SCARs caused by cephalosporins. Here, we present the first report of a polymorphism in HLA-B*15:02 in a case of DRESS syndrome induced by ceftazidime. In particular, the HLA-B*15:02 allele is highly correlated with Stevens-Johnson syndrome/toxic epidermal neurolysis (SJS/TEN) triggered by carbamazepine, oxcarbazepine, and phenytoin in some Asian populations, including Chinese, but not other SCARs (Kloypan et al., 2021). U.S. Food and Drug Administration (FDA) and Clinical Pharmacogenetics Implementation Consortium (CPIC) recommend screening the HLA-B*15:02 allele in these populations before initiation of the therapy (Kloypan et al., 2021). The HLA-B*15:02 allele is largely absent in not Asian ethnic groups such as Caucasians, African-Americans, and Hispanics (Dean et al., 2012). Therefore, HLA-B*15:02 could serve as a specific risk marker for Chinese patients susceptible to ceftazidime-induced DRESS syndrome.

### Limitation

There are two limitations in our current report. Firstly, while the official IPD-IMGT/HLA Database (http://www.ebi.ac.uk/ipd/imgt/hla/allele.html) lists 38,909 HLA and related alleles, we were only able to test two alleles due to technological constraints in genotyping. So, we need to explore more HLA alleles, especially the linkage disequilibrium alleles to provide more information. This limited scope necessitates a broader exploration of HLA alleles, particularly those exhibiting linkage disequilibrium, to provide a more comprehensive understanding. Secondly, beyond HLA alleles, we must also delve deeper into other potential risk factors, such as underlying diseases, liver and kidney function, and cytokines. Previous case studies have shown that patients with immune system disorders, including adult-onset immunodeficiency (Nie et al., 2022), Still’s disease (Saper et al., 2022), and rheumatic diseases (Adwan, 2017), tend to have inherent hypersensitivity. As such, these underlying diseases may serve as valuable predictors. To further advance our understanding, we propose analyzing published reports to ascertain disease diagnoses in each case and paying particular attention to immune system diseases. Additionally, given the reported association between cytokine-like IL-1 and IL-6 inhibitors and HLA-DRB1*15 alleles in DRESS cases with Still’s disease, we should also explore the role of cytokines in these adverse reactions (Saper et al., 2022). In summary, a more comprehensive exploration of HLA alleles, underlying diseases, and cytokines could provide valuable insights into the mechanisms and predictors of adverse reactions to medications.

## Conclusion

In conclusion, we present the first discovery of a polymorphism in HLA-B*15:02 in a case of DRESS syndrome induced by ceftazidime. This clinical observation highlights the importance of distinguishing DRESS and other allergic reactions when administering ceftazidime or other beta-lactam antibiotics. Given the potential significance of this association, further exploration of the relationship between HLA genetic types and DRESS in ceftazidime-treated patients is warranted. Additionally, utilizing pre-treatment HLA analysis as a preventative measure to identify patients at risk of developing ceftazidime-related DRESS may be a valuable option to consider in the near future.

## Data Availability

The original contributions presented in the study are included in the article, further inquiries can be directed to the corresponding authors.
